# Effects of 12-Week Anti-Inflammatory Dietary Education on Depressive Symptoms Among Depressed Patients with Breast Cancer Undergoing Adjuvant Chemotherapy: A Randomized Controlled Trial

**DOI:** 10.3390/nu17060957

**Published:** 2025-03-09

**Authors:** Lan Cheng, Yue Chen, Jianyun He, Xinxin Cheng, Yuting Wang, Xiaoxia Lin, Zhenzhen Huang, Xinyi Miao, Shufang Xia

**Affiliations:** Wuxi School of Medicine, Jiangnan University, Wuxi 214122, China; chenglan@stu.jiangnan.edu.cn (L.C.); chenyue@stu.jiangnan.edu.cn (Y.C.); hejianyun@stu.jiangnan.edu.cn (J.H.); chengxinxin@stu.jiangnan.edu.cn (X.C.); wangyuting@stu.jiangnan.edu.cn (Y.W.); linxiaoxia@stu.jiangnan.edu.cn (X.L.); 6242807010@stu.jiangnan.edu.cn (Z.H.); 6242807017@stu.jiangnan.edu.cn (X.M.)

**Keywords:** breast cancer, depressive symptoms, anti-inflammatory diet, inflammation, education

## Abstract

**Background**: Depressive symptoms (DepS) are prevalent among patients with breast cancer. Offering an anti-inflammatory diet is a promising strategy for DepS management, but it is costly and difficult to scale up. Instead, anti-inflammatory dietary education is cost-effective and may be more conducive to the promotion of an anti-inflammatory diet strategy. **Methods**: A prospective, assessor-blinded, two-arm randomized controlled trial was designed to determine the effects of 12-week anti-inflammatory dietary education on DepS in breast cancer patients with depression. Adult female patients with depression and receiving adjuvant chemotherapy were recruited. Participants in the intervention group received anti-inflammatory dietary education, while the control group received routine nursing care. Outcomes included the Center for Epidemiologic Studies Depression Scale (CES-D) score, energy-adjusted dietary inflammatory index (E-DII), plasma inflammatory biomarkers, and quality of life (QoL), which were all assessed at baseline and after a 12-week follow-up. The robustness of the estimates was investigated through sensitivity analyses. A post hoc power analysis was conducted to establish the observed effect sizes for the primary outcomes. **Results**: A total of 88.6% (62/70) of the participants completed the entire 12-week follow-up. No statistically significant between-group differences were found in the baseline characteristics, including sociodemographic factors, disease-related characteristics, and lifestyle factors. After the intervention, both the CES-D score (*p* = 0.040) and E-DII (*p* < 0.001) in the intervention group were significantly lower than in the control group, while the QoL was significantly increased (*p* < 0.001). Compared with the baseline, the tumor necrosis factor-α (TNF-α) (*p* = 0.002) and C-reactive protein (CRP) (*p* = 0.045) levels were significantly lower in the intervention group but not in the control group. **Conclusions**: Anti-inflammatory dietary education may improve DepS and QoL in breast cancer patients with depression and undergoing chemotherapy by regulating inflammation. Given its acceptability and practicality, this strategy may be incorporated into routine cancer care.

## 1. Introduction

Breast cancer has become a global health concern due to its high mortality and morbidity, with 2.3 million new cases and 665,684 deaths in 2022 [1]. Due to the life-threatening and traumatic nature of cancer diagnosis and treatment, breast cancer patients are highly susceptible to mental disorders [2], and depressive symptoms (DepS) are common in breast cancer patients, with a global prevalence of 30.2% [3], usually peaking during adjuvant therapy and rebounding after the completion of treatment [4]. Cancer-related DepS not only exacerbates physical symptoms such as fatigue, pain, and insomnia, but also leads to decreased treatment adherence, severely impaired quality of life (QoL) [5], and even a higher risk of suicide [6]. In addition, depression has been proven to be an independent predictor of breast cancer recurrence and survival [7]. Studies have shown that early preventive interventions for cancer-related DepS during oncological treatment appear to improve their QoL, prognosis, and survival rates [8]. Medication, the most common antidepressant strategy, may decrease the effectiveness of anti-cancer treatments [4], and the high costs and side effects make it difficult to achieve satisfactory outcomes. Therefore, there is an urgent need for more cost-effective interventions for breast cancer-related DepS that have fewer side effects and do not interfere with oncology therapy.

A large number of studies have shown that the primary determinants of breast cancer-related DepS encompass sociodemographic factors (e.g., age, marital status, education level, and monthly income), disease-related characteristics (e.g., cancer stage, type of treatment, and surgery), and lifestyle factors (e.g., physical activity, smoking, and alcohol use) [9,10,11,12,13]. The diet, as a safe and modifiable lifestyle behavior, has also attracted extensive attention from researchers in recent years [14]. For instance, improving an individual’s dietary behaviors, such as adopting a healthy dietary pattern that includes the consumption of vegetables, fish, olive oil, and grains, can enhance their overall mental health and reduce the risk of DepS [15,16]. A systematic review examined the association between dietary habits and depression and found that both following a healthy diet—in particular, incorporating vegetables and fruits—and avoiding a pro-inflammatory diet, consisting of junk foods, fast foods, and high meat intake, may lower the risk of developing DepS or clinical depression [17]. A meta-analysis that included 16 randomized controlled trials (RCTs) found that dietary interventions such as individualized dietary counseling, group dietary classes, and standardized dietary prescription were effective in improving DepS across the population [18]. The ‘SMILES’ RCT, which included 56 adults with major depressive disorder (MDD), showed significant improvements in depression in patients who received 12 weeks of personalized dietary advice and nutritional counseling support compared to a control group who received social support [19]. Similarly, another RCT including 152 adults with depression showed that a 6-month Mediterranean-style dietary intervention (including interactive nutrition education, cooking workshops, and fish oil supplements) improved depression to a greater extent than social support [20]. Therefore, dietary interventions are expected to be an effective way to prevent and treat DepS.

However, most breast cancer patients lack professional dietary guidance after the disease diagnosis [21] and exhibit unhealthy dietary patterns, poor dietary quality, and insufficient nutrient intake, which may be associated with an increased risk of DepS [22]. We previously found that the intake of dietary fiber, protein, some vitamins and minerals, and tryptophan was negatively correlated with DepS scores in breast cancer patients, and poor dietary quality was associated with an increased risk of DepS [23]. Accumulating evidence suggests that the diet has anti-inflammatory or pro-inflammatory potential and that its overall regulation of systemic inflammation may influence DepS [24]. Moreover, breast cancer patients are susceptible to chemotherapy side effects during adjuvant chemotherapy, and they prefer pro-inflammatory foods, leading to higher overall dietary inflammatory potential [25]. Breast cancer patients with depression have been reported to consume a pro-inflammatory diet (as assessed by a higher energy-adjusted dietary inflammatory index (E-DII) score) [26]. It has been shown that a pro-inflammatory diet is independently associated with an increased risk of DepS, especially in women, suggesting that an anti-inflammatory diet might be a potential strategy for the management of DepS [27]. In the general population, an umbrella review of 28 meta-analyses of prospective studies revealed that healthy dietary patterns and the consumption of foods with anti-inflammatory properties alleviated the risk of depression, as well as reducing DepS [28]. However, to date, no clinical trials have been conducted to explore the effects of an anti-inflammatory dietary intervention on DepS among patients with breast cancer. In our previous investigation, we found that breast cancer patients lacked knowledge about anti-inflammatory/pro-inflammatory diets and had difficulty adjusting to a healthy diet on their own. Of note, offering an anti-inflammatory diet may be a promising strategy for DepS management, but it is costly and difficult to scale up. Instead, dietary education is an important and useful tool in influencing a patient’s nutritional status [29]; it may be more cost-effective and achievable for the promotion of an anti-inflammatory diet strategy.

Therefore, the present study aimed to examine the effectiveness of 12-week anti-inflammatory dietary education in improving DepS among breast cancer patients with depression who underwent adjuvant chemotherapy. We hypothesized that, compared with the control group, patients in the intervention group would have (1) a greater improvement in DepS, (2) a greater reduction in inflammatory biomarkers, and (3) a greater improvement in QoL across the study period.

## 2. Materials and Methods

### 2.1. Study Design and Participants

This 12-week, prospective, assessor-blinded, and two-arm RCT (China Clinical Trials Registry, ChiCTR2200064967) was conducted in accordance with the Consolidated Standards of Reporting Trials (CONSORT) statement guidance [30]. Ethical approval was granted by the Medical Ethics Committee of Jiangnan University (JNU20220606IRB15) and implemented in compliance with the Declaration of Helsinki.

Female patients were recruited from October 2022 to June 2023 at the Department of Breast Surgery in the Affiliated Hospital of Jiangnan University. The inclusion criteria were (1) diagnosed with stage I~III breast cancer; (2) with a Center for Epidemiologic Studies Depression Scale (CES-D) score ≥ 16; (3) aged ≥ 18 years; (4) had undergone breast surgery and were scheduled to receive a minimum of four cycles of adjuvant chemotherapy; (5) had a smartphone to receive messages and communicate; (6) had normal cognitive function and reading abilities. The exclusion criteria included (1) recurrent or metastatic breast cancer; (2) previously diagnosed with other cancers; (3) comorbidities with serious somatic or organic brain diseases; (4) the presence of serious infectious diseases; (5) the use of antidepressant, anxiolytic, neurological, or psychiatric medication; (6) undergoing psychological treatments; (7) the recent use of antibiotics or immune-modulating medications; (8) being pregnant or lactating.

### 2.2. Recruitment and Randomization

The nursing staff identified potential participants from the medical records and then sent them invitations. Written informed consent was obtained from patients if they were eligible and interested in participating. A research assistant who did not participate in the recruitment process used the R software (version 4.3.2) to generate the randomization list with a 1:1 allocation ratio (block size of 4) and sealed the results in serially numbered opaque envelopes. Each envelope was opened only after baseline assessment completion. Based on the order of enrollment and random codes in the envelopes, participants were randomly assigned to either the intervention or control group before chemotherapy. The randomization schedule and coding of group allocations were not, at any time, accessible to the outcome assessors. After randomization, three well-trained interveners provided hospital-based anti-inflammatory dietary education and invited the participants to join a WeChat group for online nutritional counseling support. Due to the behavioral nature of the dietary intervention, the blinding of the participants and interveners was not feasible. However, several strategies were employed to reduce the risk of bias: (1) upon admission to the hospital, we firstly assessed the patient’s DepS and assigned the patients with depression to wards with patients without depression, ensuring that there would be at most one patient with depression in each ward; (2) the outcome assessors received separate training, emphasizing neutrality; (3) all assessments were conducted in dedicated rooms without intervention materials; (4) the laboratory technicians analyzing the inflammatory biomarkers were masked to the group allocations using anonymized sample IDs.

### 2.3. Sample Size

The sample size was calculated by using the following formula:

$$n={(Z}_{\alpha }+Z_{\beta }{)}^{2}\times 2\sigma^{2}/\delta^{2}$$

The difference in the mean CES-D scores of patients in the intervention and control groups *δ* = 8.1, and the combined standard deviation of the two groups *σ* = 9.34. Therefore, a minimum of 28 participants for each group was required to provide the study with 80% power at a two-tailed 5% level of significance. Considering the 20% sample attrition rate, the required sample size was increased to 70 participants, with 35 in each group.

### 2.4. Intervention

The intervention included four sessions of hospital-based anti-inflammatory dietary education and 12 weeks of online nutritional counseling support. After the patients with breast cancer were admitted to receive chemotherapy, four sessions of dietary education were provided. The aim of these sessions was to teach the participants anti-inflammatory dietary knowledge and facilitate their home-based practice. After patients were admitted to the hospital, the trained interveners provided face-to-face teaching on how to consume an anti-inflammatory diet among the participants in the ward. They were encouraged to adhere to an anti-inflammatory dietary plan characterized by the reduced consumption of processed foods and increased consumption of “good” fats and whole foods [31]. The whole foods promoted for moderate consumption included lean meats, eggs, and dairy products, while those promoted for higher consumption were fish, fruits, vegetables, nuts, and seeds. “Good fats” included monounsaturated fats with a favorable ω-6–ω-3 ratio, such as fish, seeds, and olive oil. Meanwhile, the participants were asked to limit their consumption of highly processed and refined foods, such as refined carbohydrates (pasta, bread, rice), confectionary, and processed meats. Each session lasted about 20–30 min. Before the intervention began, we prepared a brochure on the anti-inflammatory diet, including the following topics: (1) the relationship between anti-inflammatory diets and disease; (2) anti-inflammatory dietary recipes and samples of common processed foods, “good” fats, and whole foods; (3) routine care during chemotherapy; and (4) tips for achieving dietary self-management. At the end of the first dietary education session, the participants were provided with the brochures to refer to when they went home to prepare their diets. During the home period, the participants were provided with online nutritional counseling support once a week via WeChat or by telephone, which included providing dietary knowledge or nutritional tips, answering participants’ questions, reminding the participants to keep dietary records, evaluating the reasonableness of the diets, and giving targeted recommendations for improvement.

Participants in the control group received 12-week routine care, including face-to-face counseling during hospitalization (4 sessions) and online support via WeChat or by telephone after discharge if necessary. The face-to-face counseling included topics such as general knowledge of breast cancer and frequently asked questions (e.g., the occurrence, risk factors, and treatment of breast cancer; care during chemotherapy; the prevention of lymphedema; and regular review after the completion of therapy). Each of the face-to-face sessions focused on one of the selected topics. The information used in the consultation sessions was developed based on the Guidelines for Breast Cancer Diagnosis and Treatment by the China Anti-Cancer Association (2024). The interveners never took the initiative to teach diet-related knowledge to the patients in the control group. If patients inquired about the diet, recommendations were provided based on the Dietary Guidelines for Chinese Residents (2022), a basic set of dietary guidelines without a specific anti-inflammatory focus. As for online support, the researchers provided the patients with friendly reminders of upcoming tests and treatment appointments and answered patients’ questions.

To minimize the dropout rates, this study recruited breast cancer patients scheduled to receive at least four cycles (21 days per cycle) of adjuvant chemotherapy. This ensured that all patients were still at a stage requiring hospitalization for chemotherapy after the 12-week intervention. One or two days before each chemotherapy cycle, the interveners made a phone call to provide a friendly reminder of the upcoming appointment. Additionally, patients were offered prizes for completing each session of the intervention, encouraging their attendance at future follow-up visits.

### 2.5. Sociodemographic and Clinical Information

The participants’ sociodemographic and clinical characteristics were collected at baseline. As both physical activity and drinking are critical factors affecting depression [32,33], they were also assessed at baseline. The physical activity level was assessed by the International Physical Activity Questionnaire Short Form (IPAQ-SF) to calculate the metabolic equivalent (MET) and then classified into three levels: low (<600 MET/min/week), moderate (600 to 1500 MET/min/week), and high (>1500 MET/min/week) [34]. Data were collected by trained phlebotomists, dieticians, nutritionists, and research assistants.

### 2.6. Primary Outcome

The CES-D scale, with good validity in breast cancer patients, was used to assess DepS. Participants with a CES-D score ≥ 16 were considered to have depression [35,36] and selected as research candidates. At the end of the 12 weeks of intervention, the participants were reassessed with the CES-D.

### 2.7. Secondary Outcomes

#### 2.7.1. Dietary Intake Assessment and E-DII Calculation

For each intervention cycle, dietary intake was assessed using 3-day, 24 h dietary recalls [37], in which detailed types, amounts, and cooking methods of foods were recorded on one weekend day and two working days. In order to improve the accuracy of the dietary surveys, we collected the participants’ dietary intake in the past 24 h after their admission to the hospital, and the remaining two dietary recalls were conducted via WeChat after the participants were discharged from the hospital and the chemotherapy-induced gastrointestinal symptoms had disappeared. After each dietary survey, daily nutrient intakes were then calculated using the Nutrition Calculator v2.7.8.8 (Chinese Center for Disease Control and Prevention, Beijing, China). Meanwhile, the dietitian evaluated the participants’ dietary and nutrient intake roughly, gave individualized recommendations for adjustments, and educated the participants via WeChat.

To control for the effect of total energy intake on the DII score, the E-DII score was calculated to assess the overall inflammatory potential of an individual’s diet. The detailed calculation methods for the E-DII have been described in our previously published literature [26]. In this study, the E-DII scores were calculated using the following 25 food parameters: energy; total fat; protein; carbohydrates; dietary fiber; cholesterol; vitamins A, B1, B2, B6, C, D, and E; folate; niacin; β-carotene; magnesium; iron; zinc; selenium; saturated fatty acids; monounsaturated fatty acids; polyunsaturated fatty acids; and ω-3 and ω-6 fatty acids.

#### 2.7.2. Plasma Inflammatory Biomarkers

After an overnight 12 h fast, blood samples were collected from 35 volunteer participants at baseline and week 12 by a trained phlebotomist. Plasma was prepared by centrifugation at 3500 rpm for 10 min, followed by inflammatory biomarker (interleukin (IL)-1β, IL-6, tumor necrosis factor-α (TNF-α), and C-reactive protein (CRP)) measurements using commercial ELISA kits (Meimian, Yancheng, China) for each time point according to the manufacturer’s instructions.

#### 2.7.3. Quality of Life

QoL was assessed using the Functional Assessment of Cancer Therapy—Breast (FACT-B) [38], which is a scale designed specifically for breast cancer patients. It consists of a general scale measuring the commonality of all cancer types (commonality module) and breast cancer-specific domains, covering physical, social/family, emotional, functional well-being, and additional breast cancer concerns. All items were scored on a 5-point Likert scale from 0 to 4 (“not at all” to “very much”), with a total score of 0 to 144. A higher total score represents better QoL. The Chinese version of the scale has been widely validated in breast cancer patients [39].

### 2.8. Statistical Analysis

Statistical analyses were performed on SPSS 27.0 (IBM Corp, Chicago, IL, USA). The normality of continuous variables was assessed using the Shapiro−Wilk test. Continuous variables were presented as the mean ± standard deviation (SD). Differences between groups were assessed by the 2-tailed independent-samples *t*-test for normally distributed variables or the Mann−Whitney U test for non-normally distributed variables. The paired-sample *t*-test and Wilcoxon test were used to assess the differences before and after the intervention for normally and non-normally distributed data, respectively. Categorical variables were presented as frequencies (n) and percentages (%). The chi-squared test, continuity-corrected chi-squared test, or Fisher’s exact test was used to assess the differences in categorical variables between groups. The robustness of estimates was investigated through sensitivity analyses by comparing the baseline characteristics and outcomes of the subjects who consented to provide blood samples (summarized in Supplementary Materials). To aid in the interpretation of the primary outcomes, a post hoc power analysis was additionally conducted based on the observed effect sizes. A *p*-value of <0.05 was considered statistically significant.

## 3. Results

### 3.1. Overview

**Figure 1:**
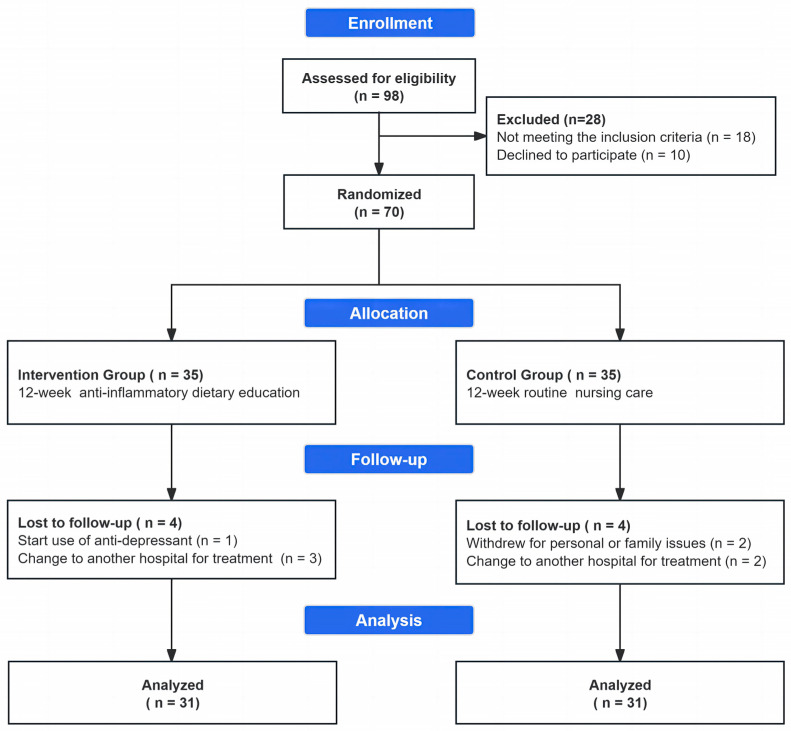
Flow diagram of participant recruitment during the trial according to CONSORT.

A total of 98 female breast cancer patients were assessed for eligibility, of whom 18 did not meet the inclusion criteria and 10 declined to participate (Figure 1). Finally, 70 eligible participants were successfully recruited and randomly assigned to the intervention group (*n* = 35) or the control group (*n* = 35). At the endpoint, a total of 62 participants (31 in each group, with a retention rate of 88.6%) completed the entire 12-week follow-up, as eight dropped out (dropout rate 11.4%, *n* = 1 due to starting the use of antidepressants, *n* = 5 due to hospital changes, and *n* = 2 due to personal or family issues).

### 3.2. Participant Baseline Characteristics

The baseline characteristics of the participants are presented in Table 1. All female participants had a mean age of 52.90 ± 11.05 years, with a mean BMI of 24.18 ± 3.22 kg/m^2^. The majority of the participants were post-menopausal (54.8%), diagnosed with stage II breast cancer (61.2%), and had undergone a mastectomy (67.7%). No statistically significant between-group differences were found in the baseline characteristics.

### 3.3. Effects on Depressive Symptoms

**Figure 2:**
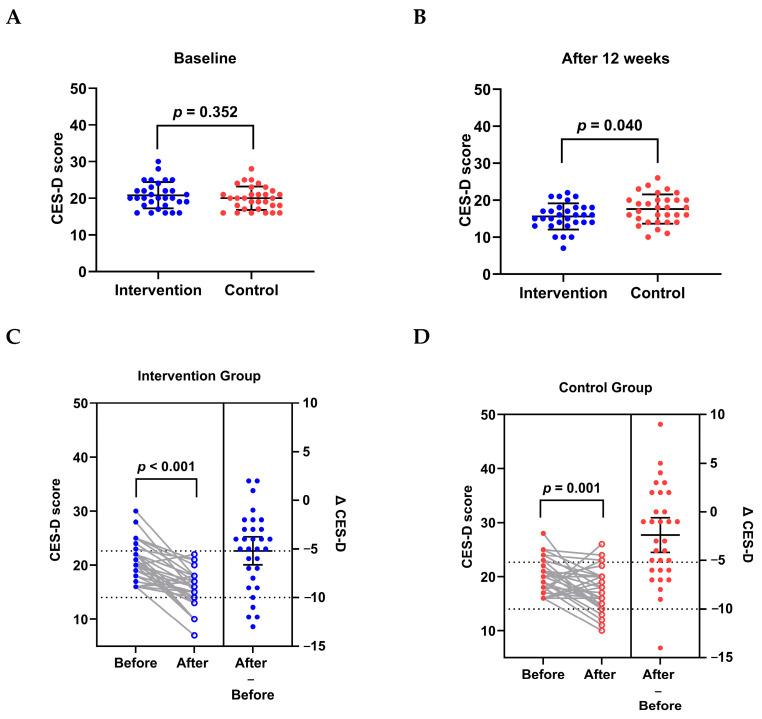
Effects on depressive symptoms in breast cancer patients with depression and receiving chemotherapy. (**A**,**B**): CES-D scores of intervention and control groups at baseline and after 12 weeks. Data are presented as mean ± SD. Independent-samples *t*-test was used. (**C**,**D**): CES-D scores of intervention and control groups from baseline to the end of follow-up. Paired *t*-test was used. CES-D, the Center for Epidemiologic Studies Depression Scale. The change (Δ) was defined as the value after the intervention minus the value at baseline for the same individual.

At baseline, the CES-D score (20.81 ± 3.59 vs. 20.00 ± 3.17; *p* = 0.352) was not significantly different between the two groups (Figure 2A). However, after the 12-week follow-up, the CES-D score in the intervention group was significantly lower than in the control group (15.61 ± 3.55 vs. 17.61 ± 3.95; *p* = 0.040; Figure 2B). Compared to the baseline data, a reduction in the CES-D scores was observed in both the intervention (Δ CES-D: −5.19 ± 3.94; *p* < 0.001; Figure 2C) and control (Δ CES-D: −2.39 ± 4.84; *p* = 0.001; Figure 2D) groups. In addition, a significant difference was also observed in the changes in the CES-D score between the two groups (*p* = 0.015).

### 3.4. Effects on Dietary Inflammatory Potential

**Figure 3:**
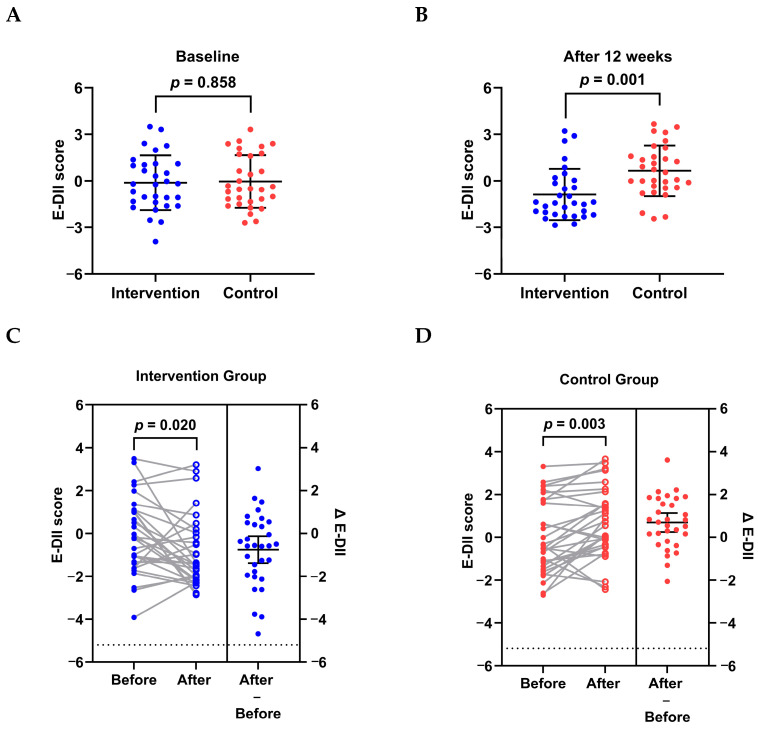
Effects on E-DII in breast cancer patients with depression and receiving chemotherapy. (**A**,**B**): E-DII scores of the intervention and control groups at baseline and after 12 weeks. Data are presented as mean ± SD. (**A**) Independent-samples *t*-test was used. (**B**) Mann−Whitney U test was used. (**C**,**D**): E-DII score changes of the intervention and control groups from baseline to the end of follow-up. Paired *t*-test was used. E-DII, energy-adjusted dietary inflammatory index. The change (Δ) was defined as the value after the intervention minus the value at baseline for the same individual.

At baseline, no significant difference was found in the E-DII scores between the two groups (*p* = 0.858; Figure 3A). However, after the 12-week follow-up, the E-DII score in the intervention group was significantly lower than in the control group (−0.88 ± 1.65 vs. 0.65 ± 1.63; *p* = 0.001; Figure 3B). Compared to the baseline data, the E-DII score was significantly decreased in the intervention group (Δ E-DII: −0.76 ± 1.71; *p* = 0.020; Figure 3C), while it was significantly increased in the control group (Δ E-DII: 0.69 ± 1.21; *p* = 0.003; Figure 3D). A significant difference was also observed in the changes in the E-DII score between the two groups (*p* < 0.001).

### 3.5. Effects on Plasma Inflammatory Biomarkers

Since some patients had already received blood tests during their pre-admission outpatient visits, only 35 plasma samples were obtained from the patients for the determination of inflammatory biomarkers. Sensitivity analyses showed that the baseline characteristics of these 35 individuals were consistent with those of the 62 individuals and that the intervention had a similar effect on the study outcomes (summarized in Supplementary Materials). As shown in Table 2, there were no significant differences in all plasma inflammatory biomarkers between the two groups at baseline and at the end of the 12-week follow-up (*p* > 0.05). Compared with the baseline data, both the TNF-α (Δ TNF-α: −11.58 ± 15.27 pg/mL; *p* = 0.002) and CRP (Δ CRP: −0.98 ± 1.80 mg/L; *p* = 0.045) levels decreased significantly in the intervention group, but not in the control group. Additionally, there was a significant difference in Δ TNF-α between the two groups (Δ TNF-α: −11.58 ± 15.27 vs. −5.29 ± 21.02; *p* = 0.041).

### 3.6. Effects on QoL

The changes in the QoL of the participants are shown in Table 3. There were no significant differences between the two groups at baseline in the FACT-B scores (*p* > 0.05). After the 12-week follow-up, the FACT-B score of the intervention group was significantly higher than that of the control group (*p* < 0.001). Although both groups experienced improved QoL following the 12-week follow-up, the intervention group showed a greater increase than the control group (Δ FACT-B: 16.77 ± 18.68 vs. 4.10 ± 17.17; *p* = 0.007). Moreover, at the end of the 12-week follow-up, the subscale scores of social/family (20.00 ± 4.82 vs. 17.10 ± 3.35; *p* = 0.015), emotional (18.39 ± 3.24 vs. 16.23 ± 3.68; *p* = 0.016), functional well-being (19.26 ± 4.41 vs. 15.39 ± 4.32; *p* < 0.001), and additional concerns (29.06 ± 3.26 vs. 26.84 ± 3.49; *p* = 0.012) were significantly higher in the intervention group than in the control group, but no significant difference was found in physical well-being.

## 4. Discussion

In this study, we examined the effectiveness of 12-week anti-inflammatory dietary education on DepS among breast cancer patients undergoing adjuvant chemotherapy. Patients who completed the dietary education showed significant improvements in DepS, dietary anti-inflammatory potential, and QoL compared to the control group. Moreover, we also found that this strategy played an important role in reducing the levels of inflammatory biomarkers (TNF-α and CRP) in breast cancer patients. Therefore, anti-inflammatory dietary education may be an effective strategy in managing DepS and improving QoL among breast cancer patients in clinical settings.

Numerous studies have indicated a positive impact of dietary interventions on the treatment of patients with depression [40]. We conducted this prospective study in breast cancer patients with depression and found that the 12-week anti-inflammatory dietary education was not only effective in enhancing the dietary anti-inflammatory potential but also exerted a beneficial effect on DepS, which was consistent with the findings of some previous studies. A systematic review and meta-analysis including seven longitudinal and four cross-sectional studies reported a significant association between a pro-inflammatory diet and an increased risk of DepS relative to an anti-inflammatory diet [41]. A randomized controlled trial including 67 adults with MDD found that, after 12 weeks of follow-up, the participants in the modified Mediterranean diet (a typical anti-inflammatory dietary pattern) group showed a better improvement in DepS than those in the control group [19]. A similar effect of this dietary pattern has also been observed in postmenopausal women, wherein long-term adherence to the Mediterranean diet not only improved mood and depression but also reduced the risk of breast cancer [42]. Of note, inconsistent results were found in overweight and obese breast cancer survivors, where anti-inflammatory dietary education had no beneficial effect on DepS in the aforementioned populations [43]. Several factors might be responsible for this discrepancy. First, the inclusion criteria for the study participants were different. The baseline CES-D scores of the breast cancer survivors in the study conducted by Long et al. [43] were 3.07 ± 2.92 and 2.33 ± 2.40, indicating that nearly all breast cancer survivors in Long’s study did not have depression, but, in the present study, all participants had depression. Second, the duration of the intervention was different. Long et al. [43] assessed the effect of the intervention at 6 and 12 months, both of which were longer than 12 weeks. Meanwhile, a two-year randomized trial designed to assess the effect of the Mediterranean diet in patients with previous depressive episodes showed no differences regarding the depression recurrence risk, but an improvement in DepS was seen at four and eight months [44]. Therefore, considering that a dietary nutrition intervention is a long-term lifestyle modification and the effects of the diet on DepS appear to undergo dynamic changes over time, future studies with more comprehensive designs, such as considering different disease stages, larger sample sizes, and longer follow-ups, are recommended to gain insights into the effects of an anti-inflammatory diet on DepS in breast cancer patients.

Inflammation is an important biological mechanism identified through which diet may plausibly affect DepS [45]. The present study revealed a notable decline in the levels of TNF-α and CRP among participants in the anti-inflammatory dietary education group, but only the change in TNF-α was significant between the intervention and control groups. This result was consistent with our previous findings that, among all determined inflammatory biomarkers (IL-1β, IL-6, TNF-α, and CRP), only TNF-α significantly mediated the relationship between the E-DII and DepS in breast cancer patients [26]. Similarly, Yao et al. [46] also found that patients with MDD had higher serum TNF-α levels, but, after 2 and 12 weeks of antidepressant treatment, there were significant decreases in their TNF-α levels. These findings indicate that depression is accompanied by the activation of TNF-α, which also has predictive value for the antidepressant treatment response in patients with depression. In breast cancer survivors, a high-quality diet that is typically rich in fruits, vegetables, whole grains, and polyunsaturated fatty acids has been reported to be associated with lower levels of pro-inflammatory biomarkers (e.g., CRP and TNF-α) [47]. Both of these inflammatory biomarkers have also been demonstrated to be strongly associated with DepS. A longitudinal study including 60 subjects with metabolic syndrome showed that, after six months of dietary treatment, DepS and the circulating CRP concentrations were decreased, and the decrease in DepS was significantly associated with declines in CRP [48]. A systematic review and meta-analysis of 63 studies indicated that circulating TNF and CRP were associated with DepS in cancer patients [49]. Thus, inflammation appears to be a key factor in the association between diet and DepS. However, considering the limited blood sample size and short-term follow-up, these observations are exploratory and require confirmation in future mechanistic studies.

Additionally, anti-inflammatory dietary education also resulted in a notable enhancement in overall QoL and its subscales of social/family, emotional and functional well-being, and additional concerns. Similar results were found in patients with relapsing–remitting multiple sclerosis and in overweight and obese women with knee osteoarthritis, where an anti-inflammatory dietary intervention significantly improved the participants’ QoL compared with controls [50,51]. A randomized controlled trial with 106 lung cancer patients receiving chemotherapy also showed an improvement in QoL with an anti-inflammatory diet [52]. Meanwhile, Barchitta et al. [53] performed a systematic review of nine experimental studies (including seven RCTs and two single-arm trials) and found that breast cancer survivors showed significant improvements in overall QoL and/or its subscales after receiving dietary interventions that included measures such as diet provision, dietary education, and diet counseling. However, another study showed no significant effect of an anti-inflammatory diet on the QoL of breast cancer survivors [43]. A possible explanation for this may be that the latter study had a longer intervention period, potentially masking a significant effect of the intervention on QoL in the short term [54]. Additionally, at the end of the intervention, there was no significant difference between the intervention and control groups in terms of the physical well-being domain. This finding is not surprising, as this subscale assesses patients’ physical well-being by asking whether the patients have experienced symptoms such as weakness, nausea, pain, being bothered by the side effects of treatment, feeling ill, and being forced to lie down in the past 7 days. However, during the investigation, we found that the participants in both groups had these symptoms and most of them were related to chemotherapy. Therefore, the effect of anti-inflammatory dietary education on patients’ physical well-being may be diminished by chemotherapy.

There are some limitations to this study. First, dietary intake was assessed using self-reported data collected through 3-day, 24 h dietary recalls, which may lead to recall bias. For most dietary components, unbiased reference instruments are lacking [55]. Although the food frequency questionnaire (FFQ) is commonly used to assess the long-term diets of individuals, we opted for the 3-day, 24 h dietary recall method instead. This change was necessitated by our observation that most patients’ dietary habits had changed a lot after their breast cancer diagnosis. Additionally, the dietary information collected through the FFQ encompassed both intake before and after the diagnosis, making it impossible to accurately reflect the patients’ diets during the disease stages. Second, anti-inflammatory dietary education is a lifestyle intervention that requires long-term adherence, whereas the present study was conducted for only 3 months and lacked long-term follow-up to assess the impact of the intervention on the patients. In the future, longer follow-up studies are needed to evaluate the long-term intervention effects. Third, only 35 plasma samples were obtained from the patients for the determination of inflammatory biomarkers. This was due to the fact that some patients had already received relevant blood tests during their pre-admission outpatient visits and were usually hospitalized a few days later to start chemotherapy immediately. Consequently, they were willing to complete the questionnaire but unwilling to have their blood drawn again specifically for inflammatory biomarker determination. Fourth, only IL-1β, IL-6, TNF-α, and CRP were assessed in this study. Several other important markers through which the diet affects DepS should be considered in future studies, including the anti-inflammatory cytokine IL-10, oxidative stress, the gut microbiome, and tryptophan [56]. Fifth, the absolute change in the CES-D scores and inflammatory biomarkers seemed minimal after the 12-week intervention. Considering the limited sample size, we conducted a post hoc power analysis for the primary outcome, achieving power of 54.1%, which is lower than the conventional threshold of 80%. However, it is much higher than the power values of 59 RCTs (median 0.16, IQR 0.08~0.32) in the surgical literature [57]. The conventional threshold is unattainable even for high-quality studies, particularly when considering studies with inherently small samples, such as many of those related to surgical science or rare diseases [58]. Finally, as a single-center study, the generalizability of the findings to other populations may be limited, and caution is needed when extrapolating these results. Whether similar benefits would extend to non-cancer populations or those with treatment-resistant depression remains unclear. Therefore, future prospective studies with more rigorous study designs, larger sample sizes, multi-center designs, and longer follow-up periods are still needed to determine the long-term effectiveness of anti-inflammatory dietary education on DepS in the breast cancer population.

The present study also has some implications. Although the observed effect sizes for the DepS scores and inflammatory biomarkers seemed minimal (potentially influenced by the limited sample size and short-term follow-up duration), the statistically significant changes suggest that anti-inflammatory dietary education may exert a measurable yet modest therapeutic effect on both emotional regulation and inflammatory pathways in breast cancer patients with depression. This finding is consistent with existing evidence: a meta-analysis of 16 RCTs (*n* = 45,826) similarly demonstrated small but significant antidepressant effects of dietary interventions [18], suggesting that dietary improvement could be an ideal option for low-intensity treatment or for individuals to adopt as a self-management approach to reduce DepS. In addition, most breast cancer patients exhibited unhealthy dietary patterns due to a lack of professional dietary guidance after their disease diagnosis, which is associated with an increased risk of breast cancer recurrence and mortality [21,59]. In the present study, we also found that breast cancer patients without anti-inflammatory dietary education had a significant increase in their E-DII scores during chemotherapy, while patients who received anti-inflammatory dietary education had a significant decrease. A prospective cohort study included 1064 female breast cancer survivors and found that the post-diagnosis E-DII was significantly associated with the mortality risk among breast cancer survivors [60]. Breast cancer patients consuming the most pro-inflammatory diets had a 34% higher risk of death from all causes compared with patients consuming the most anti-inflammatory diets [60]. Therefore, the findings of the present study offer further support for the need to focus on addressing poor diets (especially higher dietary inflammatory potential) in oncology practice. For instance, healthcare providers could incorporate anti-inflammatory dietary education into standard care pathways during the treatment initiation and follow-up phases, implementing serial assessments using validated depression scales (e.g., CES-D) alongside dietary adherence tracking (e.g., the consumption of anti-inflammatory/pro-inflammatory food). Where feasible, behavioral measures should be supplemented with inflammatory biomarkers (e.g., IL-6, CRP, TNF-α) to quantify the biological impact objectively. This multimodal strategy (behavioral–biochemical synergy) may enhance patient engagement through visible progress indicators, thereby sustaining healthy dietary adherence. The resultant improvement could potentially amplify conventional cancer therapies while concurrently ameliorating depressive symptomatology and improving quality of life.

## 5. Conclusions

The present study provides evidence that anti-inflammatory dietary education could improve DepS, regulate inflammation, and improve QoL in breast cancer patients with depression undergoing chemotherapy. However, future prospective studies with more rigorous study designs, larger sample sizes, multi-center designs, and longer follow-up periods are still warranted to determine its long-term effects on DepS in the breast cancer population.

## Figures and Tables

**Table 1:** Comparison of baseline characteristics between the intervention and control groups.

Variable	Total (*n* = 62)	Intervention (*n* = 31)	Control (*n* = 31)	*p*
Age (year) ^a^	52.90 ± 11.05	52.61 ± 11.91	53.19 ± 10.32	0.838
BMI (kg/m^2^) ^b^	24.18 ± 3.22	24.17 ± 2.80	24.18 ± 3.63	0.800
Menopausal status ^c^				
Pre-menopausal	28 (45.2)	14 (45.2)	14 (45.2)	1.000
Post-menopausal	34 (54.8)	17 (54.8)	17 (54.8)	
Marital status ^d^				
Married	57 (91.9)	29 (93.5)	28 (90.3)	1.000
Widowed/divorced/single	5 (8.1)	2 (6.5)	3 (9.7)	
Education level ^c^				
Primary school or lower	14 (22.6)	6 (19.4)	8 (25.8)	0.386
Middle school	27 (43.6)	15 (48.4)	12 (38.7)	
High school/secondary school	10 (16.1)	3 (9.7)	7 (22.6)	
Junior college or higher	11 (17.7)	7 (22.6)	4 (12.9)	
Employment ^c^				
Employed	14 (22.6)	9 (29.0)	5 (16.1)	0.455
Unemployed	25 (40.3)	12 (38.7)	13 (41.9)	
Retired	23 (37.1)	10 (32.3)	13 (41.9)	
Residence ^c^				
Rural areas	16 (25.8)	5 (16.1)	11 (35.5)	0.213
Towns	17 (27.4)	10 (32.3)	7 (22.6)	
Urban areas	29 (46.8)	16 (51.6)	13 (41.9)	
Family monthly income ^e^				
<2000 CNY	5 (8.1)	3 (9.7)	2 (6.5)	0.617
2000~5000 CNY	20 (32.2)	8 (25.8)	12 (38.7)	
>5000 CNY	37 (59.7)	20 (64.5)	17 (54.8)	
Number of chemotherapy cycles completed ^e^				
0	15 (24.2)	8 (22.6)	7 (25.8)	0.905
1	21 (33.9)	9 (38.7)	12 (29.0)	
2	19 (30.6)	10 (29.0)	9 (32.3)	
3	7 (11.3)	4 (9.7)	3 (12.9)	
Cancer stage ^c^				
I	12 (19.4)	8 (25.8)	4 (12.9)	0.416
II	38 (61.2)	17 (54.8)	21 (67.7)	
III	12 (19.4)	6 (19.4)	6 (19.4)	
Surgery type ^c^				
Mastectomy	42 (67.7)	20 (64.5)	22 (71.0)	0.587
Lumpectomy	20 (32.3)	11 (35.5)	9 (29.0)	
Presence of comorbidities ^c^				
No	41 (66.1)	21 (67.7)	20 (64.5)	0.788
Yes	21 (33.9)	10 (32.3)	11 (35.5)	
Physical activity level ^c^				
Low	26 (41.9)	12 (38.7)	14 (45.2)	0.607
Moderate	36 (58.1)	19 (61.3)	17 (54.8)	
Drinking status ^d^				
Never	57 (91.9)	27 (87.1)	30 (96.8)	0.351
Former/current	5 (8.1)	4 (12.9)	1 (3.2)	

Data are shown as *n* (%) or mean ± SD. ^a^ Independent-samples *t*-test. ^b^ Mann−Whitney U test. ^c^ Chi-squared test. ^d^ Chi-squared test with continuity correction. ^e^ Fisher’s exact test. BMI, body mass index; CNY, Chinese yuan.

**Table 2:** Changes in plasma inflammatory biomarkers.

Variable	Intervention (*n* = 18)	Control (*n* = 17)	*p*
IL-1β (pg/mL)			
Baseline	22.57 ± 13.01	18.40 ± 5.59	0.636 ^b^
After 12 weeks	26.46 ± 11.16	21.46 ± 12.77	0.067 ^b^
Δ IL-1β	3.89 ± 9.38	3.06 ± 12.85	0.287 ^b^
*p*	0.096 ^c^	0.795 ^d^	
IL-6 (pg/mL)			
Baseline	15.30 ± 7.34	13.17 ± 5.73	0.318 ^b^
After 12 weeks	15.02 ± 7.24	13.12 ± 4.64	0.351 ^b^
Δ IL-6	−0.28 ± 4.67	−0.05 ± 6.88	0.568 ^b^
*p*	0.744 ^d^	0.463 ^d^	
TNF-α (pg/mL)			
Baseline	59.74 ± 19.76	56.18 ± 37.52	0.110 ^b^
After 12 weeks	48.17 ± 11.31	50.89 ± 21.07	0.636 ^b^
Δ TNF-α	−11.58 ± 15.27	−5.29 ± 21.02	0.041 ^b^
*p*	0.002 ^d^	0.740 ^d^	
CRP (mg/L)			
Baseline	9.43 ± 2.28	8.94 ± 3.49	0.184 ^b^
After 12 weeks	8.45 ± 1.49	8.48 ± 1.68	0.961 ^a^
Δ CRP	−0.98 ± 1.80	−0.46 ± 2.61	0.184 ^b^
*p*	0.045 ^d^	0.943 ^d^	

Data are shown as mean ± SD. ^a^ Independent-samples *t*-test. ^b^ Mann−Whitney U test. ^c^ Paired *t*-test. ^d^ Wilcoxon test. CRP, C-reactive protein; IL-1β, interleukin 1β; IL-6, interleukin 6; TNF-α, tumor necrosis factor-α. The change (Δ) was defined as the value after the intervention minus the value at baseline for the same individual.

**Table 3:** Changes in quality of life after the 12-week follow-up.

Variable	Intervention (*n* = 31)	Control (*n* = 31)	*p*
FACT-B score			
Baseline	91.74 ± 20.44	91.71 ± 17.45	0.995 a
After 12 weeks	108.52 ± 14.22	95.81 ± 13.09	<0.001 ^a^
Δ FACT-B	16.77 ± 18.68	4.10 ± 17.17	0.007 ^a^
*p*	<0.001 ^c^	0.194 c	
Physical well-being			
Baseline	20.10 ± 4.08	20.32 ± 4.30	0.729 ^b^
After 12 weeks	21.81 ± 3.69	20.26 ± 3.94	0.116 ^a^
Δ Physical well-being	1.71 ± 5.62	−0.06 ± 5.03	0.195 ^a^
*p*	0.101 ^c^	0.944 ^c^	
Social/family well-being			
Baseline	16.71 ± 5.22	16.13 ± 5.25	0.777 ^b^
After 12 weeks	20.00 ± 4.82	17.10 ± 3.35	0.015 ^b^
Δ Social/family well-being	3.29 ± 4.18	0.97 ± 4.65	0.025 ^b^
*p*	<0.001 ^c^	0.336 ^d^	
Emotional well-being			
Baseline	13.74 ± 6.07	13.97 ± 5.38	0.877 ^a^
After 12 weeks	18.39 ± 3.24	16.23 ± 3.68	0.016 ^b^
Δ Emotional well-being	4.65 ± 5.14	2.26 ± 4.84	0.065 ^a^
*p*	<0.001 ^c^	0.014 ^c^	
Functional well-being			
Baseline	15.55 ± 5.14	14.52 ± 4.73	0.414 ^a^
After 12 weeks	19.26 ± 4.41	15.39 ± 4.32	<0.001 ^a^
Δ Functional well-being	3.71 ± 5.29	0.87 ± 5.95	0.052 ^a^
*p*	<0.001 ^c^	0.422 ^c^	
Additional concerns			
Baseline	25.65 ± 4.59	26.77 ± 4.00	0.299 ^b^
After 12 weeks	29.06 ± 3.26	26.84 ± 3.49	0.012 ^a^
Δ Additional concerns	3.42 ± 5.03	0.06 ± 3.99	0.005 ^a^
*p*	<0.001 ^c^	0.929 ^c^	

Data are shown as mean ± SD. ^a^ Independent-samples *t*-test. ^b^ Mann−Whitney U test. ^c^ Paired *t*-test. ^d^ Wilcoxon test. FACT-B, the Functional Assessment of Cancer Therapy—Breast Scale. The change (Δ) was defined as the value after the intervention minus the value at baseline for the same individual.

## Data Availability

The data presented in this study are available from the corresponding author upon request due to privacy.

## Supplementary Materials

The following supporting information can be downloaded at: https://www.mdpi.com/article/10.3390/nu17060957/s1, Table S1. Comparison of characteristics of patients who consented to provide blood samples (*n* = 35); Table S2. Changes in CES-D score, E-DII score, and quality of life of patients who consented to provide blood samples (*n* = 35).

## Abbreviations

The following abbreviations are used in this manuscript:

BMI	Body mass index
CES-D	Center for Epidemiologic Studies Depression Scale
CNY	Chinese yuan
CRP	C-reactive protein
DepS	Depressive symptoms
E-DII	Energy-adjusted dietary inflammatory index
FACT-B	Functional Assessment of Cancer Therapy—Breast Scale
IL-1β	Interleukin-1β
IL-6	Interleukin-6
TNF-α	Tumor necrosis factor-α
MDD	Major depressive disorder
MET	Metabolic equivalent
QoL	Quality of life
RCT	Randomized controlled trial
SD	Standard deviation
