# Supplementary material for: Effects of 12-Week Anti-Inflammatory Dietary Education on Depressive Symptoms Among Depressed Patients with Breast Cancer Undergoing Adjuvant Chemotherapy: A Randomized Controlled Trial
Source: Nutrients. 2025 Mar 9;17(6):957. doi: 10.3390/nu17060957 (PMC11944683; doi:10.3390/nu17060957)
Supplement: Supplementary file 1 [file nutrients-17-00957-s001.zip › nutrients-3499497-supplementary.pdf]

Supplementary materials

Table S1. Comparison of characteristics of patients who consented to provide blood samples (n = 35).

Variables	Total (n = 35)	Intervention (n = 18)	Control (n = 17)	*p*
Age (year) [a]	52.09 ± 10.95	52.00 ± 10.45	52.18 ± 11.79	0.963
BMI (kg/m^2) [a]	23.95 ± 3.47	23.80 ± 2.28	24.12 ± 4.47	0.790
Menopausal status [b]				
Pre-menopausal	13 (37.1)	7 (38.9)	6 (35.3)	0.826
Post-menopausal	22 (62.9)	11 (61.1)	11 (64.7)	
Marital status [c]				
Married	32 (91.4)	17 (94.4)	15 (88.2)	0.959
Widowed/divorced/single	3 (8.6)	1 (5.6)	2 (11.8)	
Education level [d]				
Primary school or lower	11 (31.4)	4 (22.2)	7 (41.2)	0.338
Middle school	13 (37.1)	9 (50.0)	4 (23.5)	
High school/secondary school	5 (14.3)	3 (16.7)	2 (11.8)	
Junior college or higher	6 (17.2)	2 (11.1)	4 (23.5)	
Employment [d]				
Employed	7 (20.0)	5 (27.8)	2 (11.8)	0.223
Unemployed	16 (45.7)	9 (50.0)	7 (41.2)	
Retired	12 (34.3)	4 (22.2)	8 (47.0)	
Residence [d]				
Rural areas	9 (25.7)	3 (16.7)	6 (35.3)	0.526
Towns	12 (34.3)	7 (38.9)	5 (29.4)	
Urban areas	14 (40.0)	8 (44.4)	6 35.3)	
Family monthly income [d]				
< 2000 CNY	4 (11.4)	3 (16.7)	1 (5.9)	0.785
2000 ~ 5000 CNY	11 (31.4)	5 (27.8)	6 (35.3)	
> 5000 CNY	20 (57.2)	10 (55.5)	10 (58.8)	
Number of chemotherapy cycles completed [d]				
0	11 (31.4)	5 (27.8)	6 (35.3)	0.890
1	13 (37.2)	8 (44.4)	5 (29.4)	
2	7 (20.0)	3 (16.7)	4 (23.5)	
3	4 (11.4)	2 (11.1)	2 (11.8)	
Cancer stage [d]				
I	7 (20.0)	4 (22.2)	3 (17.7)	0.742
II	18 (51.4)	8 (44.5)	10 (58.8)	
III	10 (28.6)	6 (33.3)	4 (23.5)	
Surgery type [b]				
Mastectomy	24 (68.6)	12 (66.7)	12 (70.6)	0.803
Lumpectomy	11 (31.4)	6 (33.3)	5 (29.4)	
Presence of comorbidities [b]				
No	24 (68.6)	13 (72.2)	11 (64.7)	0.632

Yes	11 (31.4)	5 (27.8)	6 (35.3)	
Physical activity level [b]				
Low	12 (34.3)	5 (27.8)	7 (41.2)	0.404
Moderate	23 (65.7)	13 (72.2)	10 (58.8)	
Drinking status [c]				
Never	32 (91.4)	16 (88.9)	16 (94.1)	1.000
Former/current	3 (8.6)	2 (11.1)	1 (5.9)	

Data are shown as n (%) or mean ± SD. [a] Independent samples t-test. [b] Chi-squared test. [c] Chi-squared test with continuity correction. [d] Fisher's exact test. BMI, body mass index; CNY, China yuan.

Table S2. Changes in CES-D score, E-DII score and quality of life of patients who consented to provide blood samples (n = 35).

Variables	Intervention (n = 18)	Control (n = 17)	*p*
**CES-D score**			
Baseline	20.83 ± 3.85	19.41 ± 3.06	0.287 [b]
After 12 weeks	14.22 ± 2.98	16.76 ± 2.95	0.016 [a]
Δ CES−D	−6.61 ± 3.63	−2.65 ± 3.82	0.009 [b]
p	< 0.001 [d]	0.011 [c]	
**E-DII score**			
Baseline	−0.50 ± 1.70	−0.04 ± 1.59	0.503 [b]
After 12 weeks	−1.33 ± 1.58	0.59 ± 1.63	< 0.001 [b]
Δ E−DII	−0.83 ± 1.32	0.63 ± 0.90	< 0.001 [a]
p	0.004 [d]	0.011 [c]	
**FACT-B score**			
Baseline	84.83 ± 13.41	87.18 ± 16.63	0.648 [a]
After 12 weeks	109.61 ± 12.56	98.12 ± 12.71	0.011 [a]
Δ FACT−B	24.78 ± 11.79	10.94 ± 12.10	0.002 [a]
p	< 0.001 [c]	0.002 [c]	

Data are shown as mean ± SD. [a] Independent samples t-test. [b] Mann−Whitney U test. [c] Paired t-test. [d] Wilcoxon test. CES-D, the Center for Epidemiologic Studies Depression Scale. E-DII, energy-adjusted dietary inflammatory index. FACT-B, the Functional Assessment of Cancer Therapy-Breast Scale. The change (Δ) was defined as the value after the intervention minus the value at baseline for the same individual.
